# Morphology, Geology and Water Quality Assessment of Former Tin Mining Catchment

**DOI:** 10.1100/2012/369206

**Published:** 2012-06-18

**Authors:** Muhammad Aqeel Ashraf, Mohd. Jamil Maah, Ismail Yusoff

**Affiliations:** ^1^Department of Chemistry, University of Malaya, Kuala Lumpur 50603, Malaysia; ^2^Department of Geology, University of Malaya, Kuala Lumpur 50603, Malaysia

## Abstract

Bestari Jaya, former tin mining catchment covers an area of 2656.31 hectares comprised of four hundred and forty-two different-size lakes and ponds. The present study area comprise of 92 hectares of the catchment that include four large size lakes. Arc GIS version 9.2 used to develop bathymetric map, Global Positioning System (GPS) for hydrographical survey and flow meter was utilized for water discharge analysis (flow routing) of the catchment. The water quality parameters (pH, temperature, electric conductivity, dissolved oxygen DO, total dissolved solids TDS, chlorides, ammonium, nitrates) were analyzed by using Hydrolab. Quality assurance (QA) and quality control (QC) procedures were strictly followed throughout the field work and data analysis. Different procedures were employed to evaluate the analytical data and to check for possible transcription or dilution errors, changes during analysis, or unusual or unlikely values. The results obtained are compared with interim national water quality standards for Malaysia indicates that water quality of area is highly degraded. It is concluded that Bestri Jaya ex-mining catchment has a high pollution potential due to mining activities and River Ayer Hitam, recipient of catchment water, is a highly polluted river.

## 1. Introduction

Each water body has an individual pattern of physical and chemical characteristics which are determined largely by the climatic, geomorphological, and geochemical conditions prevailing in the drainage basin and the underlying aquifer. Summary characteristics, such as pH, temperature, dissolved oxygen, total dissolved solids, conductivity, and redox potential, provide a general classification of water bodies of a similar nature. Mineral content, determined by the total dissolved solids present, is an essential feature of the quality of any water body resulting from the balance between dissolution and precipitation. Oxygen content is another vital feature of any water body because it greatly influences the solubility of metals and is essential for all forms of biological life.

The physical and chemical quality of the aquatic environment varies according to local geology, the climate, the distance from the ocean, and the amount of soil cover, and so forth. If surface waters were totally unaffected by human activities, up to 90–99% of global freshwaters, depending on the variable of interest, would have natural physical and chemical concentrations suitable for aquatic life and most human uses. Physical and chemical conditions in water reservoirs, such as occur in ex-mining lakes, salt lakes, hydrothermal waters, acid volcanic lakes, and peat bogs, usually make the water unsuitable for human use. Nonetheless, a range of aquatic organisms have adapted to these extreme environments.

The generation of mine water pollution, both during and after mining operations, has characterised the industry worldwide since ancient times [1–5]. In the case of acid mine drainage (AMD) waste waters, the problem may persist for many decades to thousands of years [6, 7]. These waters are generally characterised by reduced pH, elevated levels of a range of heavy metal contaminants, most notably iron, and salts such as sulphates and chlorides. The environmental consequences of mine water pollution have been comprehensively described [2, 3, 8, 9].

When mining activities are associated with the exposure of pyrite (FeS_2_) and other sulphide containing minerals, their oxidation, maybe both chemically and microbiologically mediated, has been identified as the main source of acid contamination in AMD generation [2, 5, 10–13]. AMD generation can occur in underground mine workings, waste rock dumps, mill tailings piles, ore stockpiles, spent ore piles from heap leach operations, and in other residue deposits which present a high surface area for oxidation [14]. It should be noted, however, that not all mine water is characterised by low pH and may contain elevated concentrations of metals at near neutral or alkaline pH values [9].

The importance of mine water pollution is predicated on its potential negative human health impacts and financial and environmental risks and liabilities. Globally, estimates of the impacts and the extent of the problem on various water resources have been reported for a number of regions. For example, estimates by the United States of America's Bureau of Mines indicate that over 19,000 km of rivers and streams and 73,000 hectares of lakes and reservoirs are negatively impacted by mine water from abandoned coal and metal mines [2]. The total length of watercourses negatively impacted by mine water in Europe exceeds 5000 km [9]. In the United Kingdom, an accidental discharge of 54 mL of highly acidic metal contaminated mine water into the Carnon River, from the Wheal Jane Mine in Cornwall, affected approximately 6.5 million square meters of receiving waters, with peak zinc and cadmium concentrations reaching 540 mg/L and 600 mg/L, respectively [2].

The impacts of mine water pollution on biological systems are mostly severe. The consequence of acidity and heavy metal contamination in aquatic and terrestrial ecosystems is a reduction in both species diversity and the total biomass composition of such systems [15]. Bell et al. studied a coal mine in South Africa abandoned in 1947 and found that, by 1996, the mine was still discharging AMD into an adjoining river resulting in sulphate content in excess of 1000 mg/L and pH < 3.2 [16]. This has resulted in severe adverse effects on vegetation in the surrounding area, with approximately three hectares almost completely denuded and the near total destruction of aquatic life in the seepage area.

The objectives of the investigation are to study the morphology, geology, hydrology, and physical and chemical properties of the selected lakes in the catchment in order to assess pollution impact of the lake water as it flows downstream to the Ayer Hitam River and ultimately Selangor River flowing in the catchment area.

### 1.1. The Catchment Bestari Jaya

**Figure 1:**
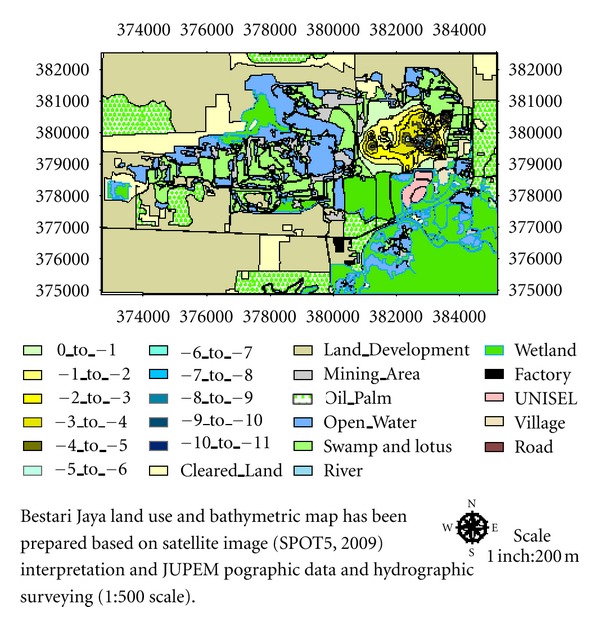
Land use map Bestari Jaya.

The study area Bestari Jaya catchment is located at 3°, 24′ 40.41′′N, and 101° 24′ 56.23′′E and is part of Daerah Kuala Selangor in Selangor state that includes three towns Mukim Batang Berjuntai, Mukim Ulu Tinggi, and Mukim Tg. karang (Figure 1). The old name of Bestari Jaya is Batang Berjuntai. In 2007, the name Batang Berjuntai was renamed “Bestari Jaya” due to censorship by the government, as “Batang Berjuntai” had phallic meanings in Malay. Bestari Jaya has a tropical, humid climate, with very little variations in temperature throughout the year. The average temperature of the area is 32°C during day and 23°C at night. An annual average rainfall is 2000 mm and 3000 mm with potential evaporation of 1600 mm per year [17].

Bestari Jaya is an old tin mining area for over 10 years. The whole catchment covers an area of 360 hectares which is located downstream at the embankment of Bestari Jaya village and University Industry Selangor (UNISEL) main campus. The catchment flow downstream to Ayer Hitam River and Udang River which ultimately ends up with Selangor River at 5 Km upstream of Batang Berjunti Water Treatment Plants (SSP1 and SSP2) which are major water distributors to federal territory (Kuala Lumpur and Putrajaya) and Selangor state as well [18].

The area consists of myriad ecosystems which can be subdivided into several categories such as degraded land, large open lakes and small ponds, earth drains and wetlands area, tin tailings (sand and slime tailings), logged peat swamp forest land in east. The contribution of storm water, peat swamp forest water, and recent sand mining activity has caused severe environmental pollution due to drainage problem in the area. The area has a lot of big lakes and small ponds that are interconnected by earth drains. Excess water from these lakes and ponds is discharged to the existing earth drains at the downstream of the lakes and ponds. Precipitation rate is high in some stagnant ponds [19].

## 2. Material and Methods

A satellite image of Bestari Jaya (SPOT, 2009) interpretation and JUPEM (Jabatan Ukur Dan Pemetaan Malaysia) porgraphic data (Scale 1 : 500) were used as a base map. GIS mapping (Arc GIS version 9.2) was used to develop final land use and bathymetric map from the base map where known points such as road junctions, bridges, milestones along trunk roads were used as reference point. Locations of the lakes were identified with reference to these known points from which a GPS (Global positioning system) survey was carried out. Hydrographical survey of the mined out lakes and ponds was carried out on a fibre glass engine boat conducted by using Garmin Fish Finder 160C and GPS (Global Positioning System), while a tape and compass were used to study the shapes and sizes of the lakes and ponds. Global Positioning System (GPS) was used to determine the actual coordinates of the surveying sites and to reconfirm the location of these sites during subsequent sampling periods. Readings were taken at 6 to 8 points in the central part of each lake to obtain the average depth. To study the thickness of the sediments depth, narrow, hollow aluminium rods were manually into the sediments. Water discharges from the catchment to Ayer Hitam river are measured by current flow meter. A commonly applied methodology for measuring and estimating, the discharge is based on a simplified form of the continuity equation [20]. The equation implies that, for any incompressible fluid, such as liquid water, the discharge (*Q*) is equal to the product of the stream's cross-sectional area (*A*) and its mean velocity ($\overset{®}{u}$) and is written as

(1) $$Q=A\overset{®}{u},$$

where *Q* is the discharge ([L^3^T^−1^]; m^3^/s or ft^3^/s), *A* is the cross-sectional area of the portion of the channel occupied by the flow ([L^2^]; m^2^ or ft^2^), $\overset{®}{u}$ is the average flow velocity ([LT^−1^]; m/s or ft/s).

The water quality parameters such as pH, temperature, electric conductivity, dissolved oxygen DO, total dissolved solids TDS, chlorides, ammonium, and nitrates were analysed by using Hydrolab MS5 USA *in situ* on a fibre glass engine boat. The Hydrolab was recalibrated every three hours or every third site, depending upon which came first. The meter electrodes were rinsed with de-ionized water before and after each measurement. Colour of water is measured by true colour units (TCU) [21], and Turbidity is measured by using electronic turbidity meter HI 98703 by HANNA Instruments Co. [22]. Turbidity is measured in nephelometric turbidity units (NTU). Total ten field works were conducted for catchment characterisation and to complete the sampling of the study area. First field work was conducted on 20th July 2009.

### 2.1. Quality Assurance and Quality Control Procedures

Quality assurance (QA) and quality control (QC) procedures were strictly followed throughout the field work and data analysis [23]. Following the applications of QA/QC procedures, the potential biases introduced by different field analysis are evaluated as they relate to the results of data analyses presented in this paper. The improvements recommended by the USEPA (2011) review resulted in QA/QC procedures that include better documentation of methods, field analysis, and better chain-of custody records. Different tests [24, 25] employed to evaluate the analytical data and to check for possible transcription or dilution errors, changes during analysis, or unusual or unlikely values.

## 3. Results and Discussion

### 3.1. Geology and Morphology of the Catchment

**Figure 2:**
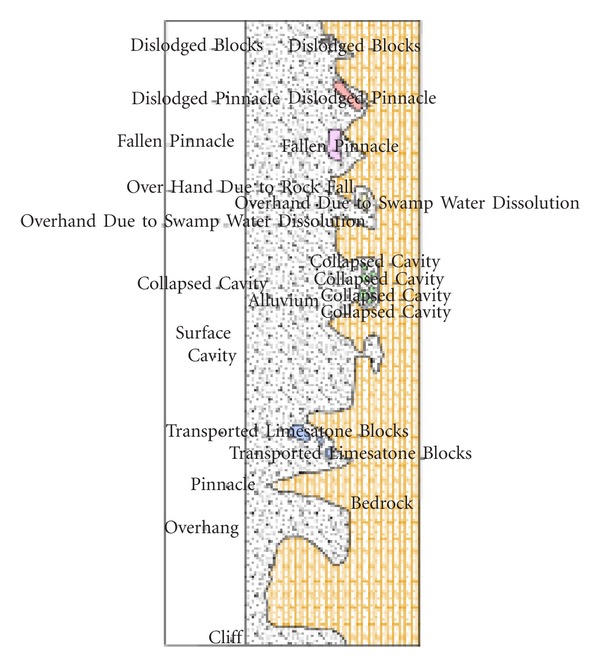
Some profiles of subsurface karstic limestone.

Tin mining at the Bestari Jaya catchment was carried out mainly in the alluvium for the rich concentration of cassiterite which was found in the catchment soil or which was trapped within the troughs of the pinnacled limestone bedrock [26]. However, in a few areas such as north east or west, mining was carried out in the residual granitic soil. Cassiterite was also mined from lodes which dipped steeply into the limestone bedrock, forming what is known as pipes. The end result of mining in almost all cases, except for the stripping of cassiterite disseminated residual soil from the hill slopes, is the formation of lakes [27] (Figure 2).

#### 3.1.1. Nature of Bedrock in Mines

The bed rock underlying the alluvium, mine tailings, and ex-mining lakes in the Bestari Jaya catchment consists mainly of limestone with interbred of schist, shale, and rarely quartzite. The most common type of bed rock found in the floor of mine pits is lime stone. The close association of rich alluvium tin deposits with limestone is the result of fortuitous combination of several geological events [28] and factors such as the style of mineralization [29], past paleoclimatic conditions [26], changes in sea levels and base levels of erosion [30], weathering characteristics of bed rock [27], and the sequence of occurrence of these events.

#### 3.1.2. Mining Methods at the Catchment

 In the past, the most common method of mining was to use the traditional gravel pumps. This method which is labour intensive was very popular with the local miners. Another widely used method was to use the capital intensive dredges. Most of these dredges were European owned. Other methods which included mining cassiterite-disseminated residual soil from hill slopes and lode mining along pipes were rare.

On most occasions, areas which were mined with dredges were often remind with gravel pumps, the reason, being the buckets in the dredges could not recover cassiterite- rich alluvium trapped within the troughs of the lime stone pinnacles.

#### 3.1.3. Ponds Resulting from Dredging Operations

**Figure 3:**
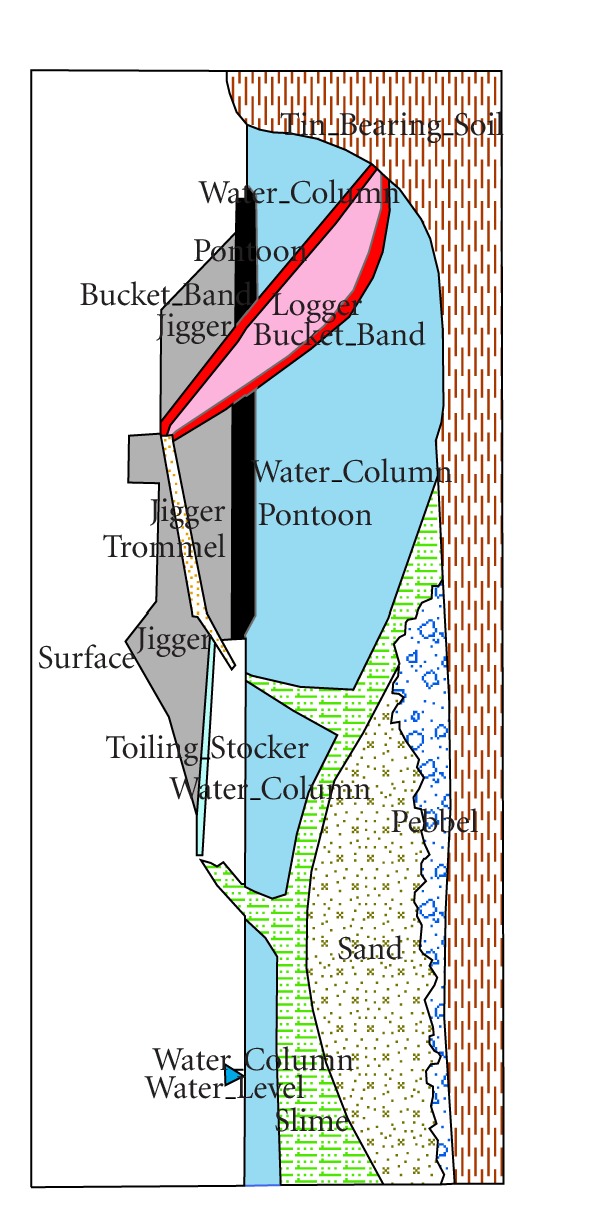
Dredge methods of mining [28].

Dredging is carried out with a dredge which comprises a mechanical excavator and a screening and washing plant mounted on a pontoon which floats on a large man-made pond (Figure 3). Cassiterite-bearing alluviums are excavated from the bottom and/or from the sides of the pond by the mechanical excavator at the front of the pontoon. The excavator which comprises a continuous chain of steel buckets scoops up the tin-bearing alluvium which are then chuted into a screen where gravels, pebbles, stiff clay balls are separated. The oversized materials are dumped back into the pond via a rock chute. The finer screened alluvium is then transferred into a sluice box where the heavier tin ore and other heavy minerals are separated from the lighter sand and clayey materials [31]. The sand and the clayed material which are known as tailings are conveyed along a stacker for dumping at the rear of the dredge. The heavier sand settles onto the pond bottom with a finer silt and clay over laying it (Figure 3).

#### 3.1.4. Ponds from Gravel Pump Mining

**Figure 4:**
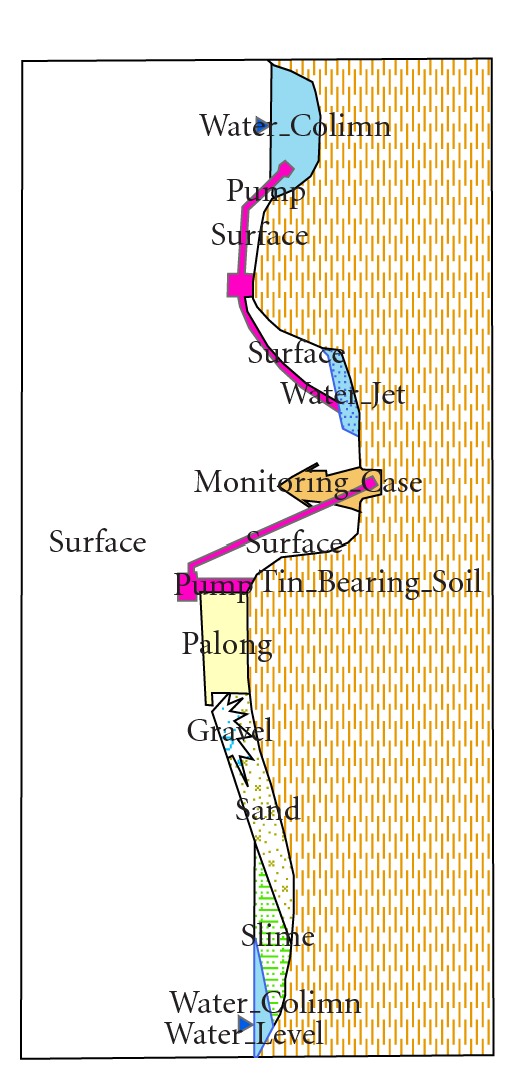
Gravel pump mining methods.

Gravel pump mining is carried out in an open pit excavated mine pit (Figure 4) where a powerful water jet monitor is used to break up the tin-bearing alluvium on the sides of the mine. The resulting slurry is washed into a sump from which it is pumped to the top of sluice box (palong). As the slurry flows down the sluice box, the heavier tin ore is separated from the gravels, sand, and clay minerals (collectively known as tailings). The tailings are deposited in a tailing pond. The coarser materials are deposited near the discharge point (Figure 4). Occasionally, the finer clay and silt are drained into a sedimentation pond for settlement, and water from this sedimentation pond is composed mostly of clay particles and is always very thick [32].

Bestari Jaya former tin mining catchment has an area of 2656.31 hectares. This vast area of the catchment has total of a 442 different-size lakes and ponds left over after different types of mining activities [33].  Table 1 shows the details of these mining activities. The selected study area of the catchment includes four big-size lakes. Selection of the area is based on its geological importance (intense mining activities, high pollution impact on the ecosystem) as the waste water flows from the area toward downstream into Ayer Hitam River and small Udang River. Both rivers end up into Selangor River. The junction has almost 5 Km distance from Batang Berjunti Water Treatment Plants (SSP1 and SSP2) which are major clean water distributors to federal territory (Kuala Lumpur and Putrajaya) and Selangor state as well [34].

Total of three lakes out of four studied lakes were left over of dredging operations. Tailing from dredging operations was deposited at the back of the dredge into the lake as such, these lakes were usually shallow except Lake A where the water depth was up to 10.0-11.0 m. Some of the lakes in the studied area were dewatered leaving behind vast stretches of tailing sand with depressions in places; these depressions formed small ponds, with some measuring about 30 to 40 m long. Some of the small ponds were aligned in a north south direction which is indicative of the orientation of the original pond. The sides of the ponds may be steep to gently dipping and are usually shallow as compared to the centre. However, there are exceptions where the centres of the ponds are shallower as compared to the sides. These ponds generally have a thin layer of slime, varying between 0.1 and 2.6 m, and a few do not even have any slime. Ponds which are formed from the palong method of mining are usually small, varying between 100 and 500 m long but a few are large, measuring up to between 700 and 1500 m long. Ponds which are relicts of former mine-pits generally have deep waters, and slime is not present at the bottom of these ponds. In fact, 52 out of the total 442 (33.3%) lakes and ponds surveyed do not have slime at the pond bottoms. Tailing ponds generally have a thin to moderately thick layer of slime (about 0.5 to 3.0 m). Sedimentation ponds, in contrast, have shallow water and thick layers of slime generally in excess of 3 m.

Survey of the lakes in the study area was carried out over a period of 6 months, from September 2009 till February 2010. As such, the water levels will be influenced by the climatic variations. Piezometers were used to check the fluctuation in water level during six months and found that lowest variation was 0.6 m and highest was 3.4 m. The average fluctuation was about 1 to 1.5 m overall for the whole year. In all piezometers, there was a marked rise in the groundwater level in the months of April to May and the conversely a drop in the months of August to September. Other than the climatic variations, local conventional rainfalls as well as local development in any one area (such as draining away of lake waters) can affect the water levels in the lakes.

#### 3.1.5. Water Depth and Discharge Analysis

**Figure 5:**
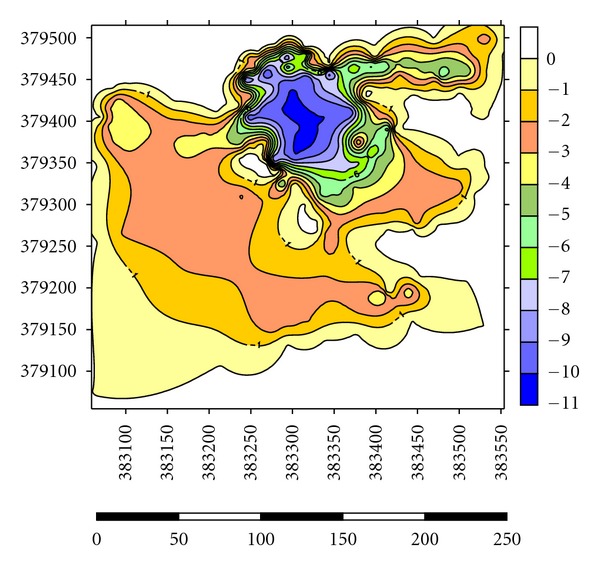
Showing depth at Lake A of the catchment.

**Figure 6:**
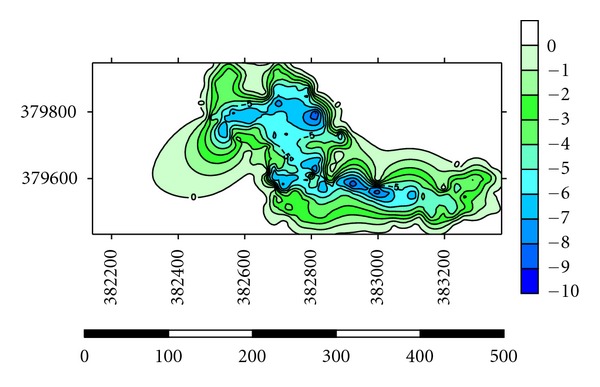
Showing depth at Lake B of the catchment.

**Figure 7:**
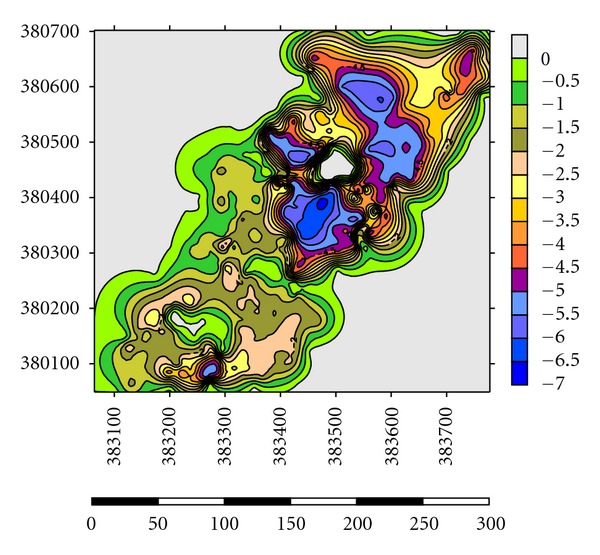
Showing depth at Lake C of the catchment.

**Figure 8:**
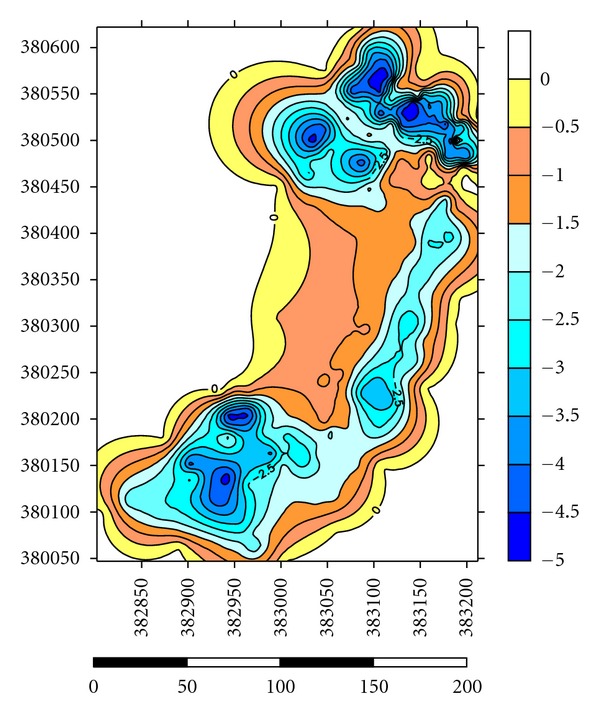
Showing depth at Lake D of the catchment.

**Figure 9:**
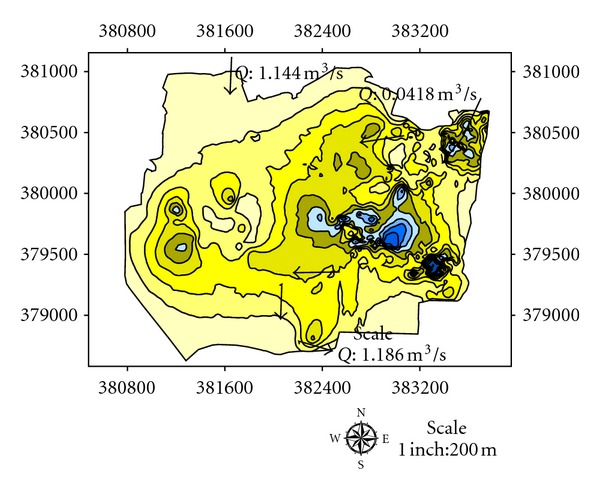
Complete map of the study area showing depth of studied lakes at the catchment.

Depths of the lakes were measured by using GPS and Garmin fish finder 160C. Lake A has the highest depth until 10.0-11.0 m at the centre where most of the dredging operations have carried out and the average depth for dredging operations is 3.0–5.0 m (Figure 5). Maximum length of the lake is 1625 m, and maximum width is 875 m. Average thickness of sediments is 1.0 m with a volume of 14500 m^3^. Side slopes of lakes are steep with undulating thin grass cover on beds (Table 2). Lake B has the highest depth until 9.0-10.0 m at the centre where most of the dredging operations have carried out and the average depth for dredging operations is 2.0-3.0 m (Figure 6). Maximum length of the lake is 1354 m, and maximum width is 689 m. Average thickness of sediments is 0.82 m with a volume of 12355 m^3^. Side slopes of lakes are moderate with undulating thin grass cover on beds (Table 2). Lake C has the highest depth until 6.0-7.0 m at the centre where most of the dredging operations have carried out and the average depth for dredging operations is 1.0-2.0 m (Figure 7). Maximum length of the lake is 1273 m, and maximum width is 791 m. Average thickness of sediments is 0.97 m with a volume of 13764 m^3^. Side slopes of lakes are gentle with undulating thin grass cover on beds (Table 2). Lake D has the highest depth until 4.0-5.0 m at the centre where most of the palong mining operations have carried out. The average depth for dredging operations is 0.5–1.0 m (Figure 8). Maximum length of the lake is 1485 m, and maximum width is 804 m. Average thickness of sediments is 0.74 m with a volume of 12355 m^3^. Side slopes of lakes are steep with undulating thin grass cover on beds (Table 2).  Figure 9 shows complete map of study area where can be seen most of the deep dredging operations are at the south eastern part of the catchment near inlet into River Ayer Hitam.

The water discharge of the catchment above a certain location is determined by the surface area of all lakes which drains toward the river from above that point. The river's discharge at that location depends on the rainfall on the catchment or drainage area and the inflow or outflow of groundwater to or from the area as well as evaporation and evapotranspiration from the area's land and plant surfaces. Average velocities and the cross-sectional area of the stream are measured for a given stream level. The velocity and the area give the discharge for that level.

**Figure 10:**
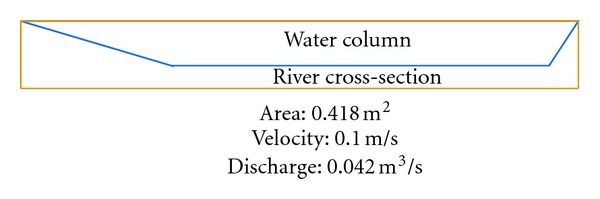
Average flow from eastern part of the catchment.

**Figure 11:**
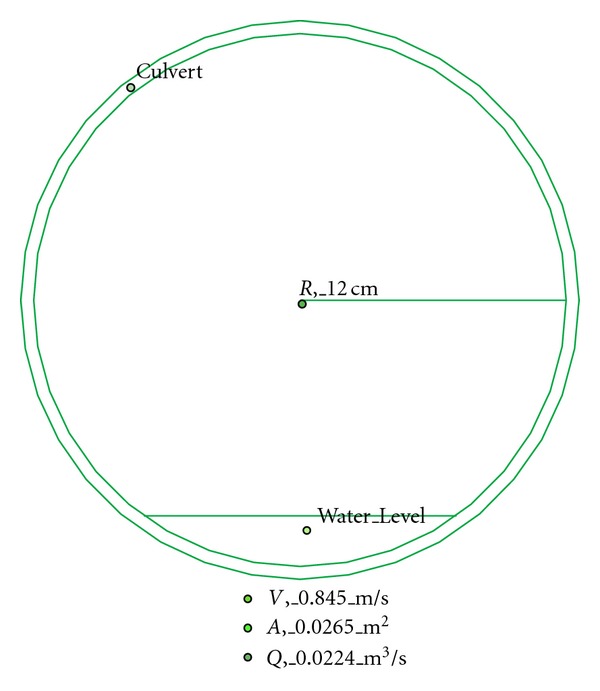
Average flow from western part of the catchment.

**Figure 12:**
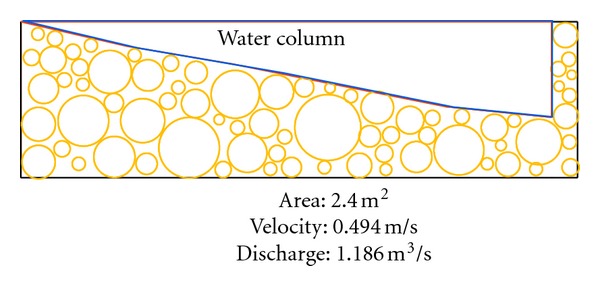
Average flow from studied lakes into River Ayer Hitam.

**Figure 13:**
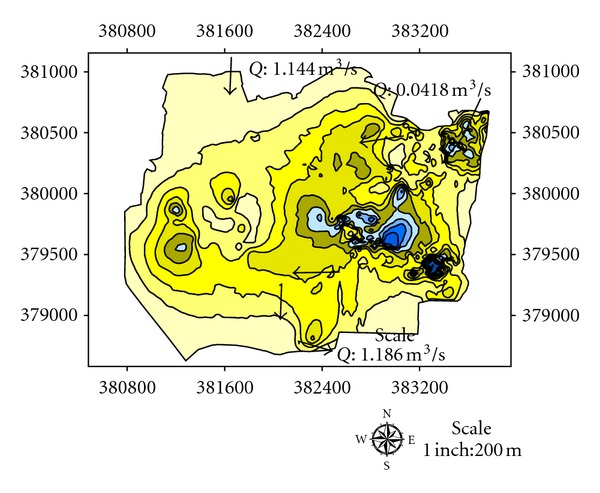
Map showing complete water discharge from catchment into River Ayer Hitam.

There are two inlets from eastern and western parts of the catchment into the southern part of the catchment area selected for our study.  Figure 10 shows that average water discharge from the eastern part of the catchment into the selected part of study area is 0.0418 m^3^/s. The western part of the catchment is connected to selected study area by an iron pipe whose inner diameter is 21 cm with average water discharge 0.0224 m^3^/s (Figure 11). There are total five inlets, namely, A, B, C, D, E from lakes of study area with average water flow 0.052 m^3^/s, 0.112 m^3^/s, 0.246 m^3^/s, 0.346 m^3^/s, and 0.430 m^3^/s, respectively (Figure 12). The measured average water discharge from these lakes into River Air Hitam is 1.186 m^3^/s.  Figure 13 shows complete water flow of the catchment into River Ayer Hitam. High water flow is an indicative of high sediment discharge from the catchment into the river as well as impact on the ecosystem. In the study area, River Selangor (recipient of water from River Ayer Hitam) has an average water depth 5.7 meter, channel width 8.4 meter, and river flow 54.6 m^3^/sec.

Ayer Hitam has an average water depth 1.7 meter, channel width 5 meter, and river flow is 21.5 m^3^/sec, while River Udang (the smallest channel) has an average water channel depth of 32 cm, channel width 110 cm and 42 m^3^/sec. The wetlands have an area of 79.7 hectors that is stretched along the north western border of the site. Several useful plant species have been seen in the wetland, while several harmful weed species have been also seen in the study area that cause blocking of water courses and water become foul due to large masses of water leaves. This area is sandy in texture, and it is representative of entire examining area in the country. The parent material is of riverine alluvium materials, with pH in range of 3.5–5.5 [34].

### 3.2. Water Quality Characteristics

**Figure 14:**
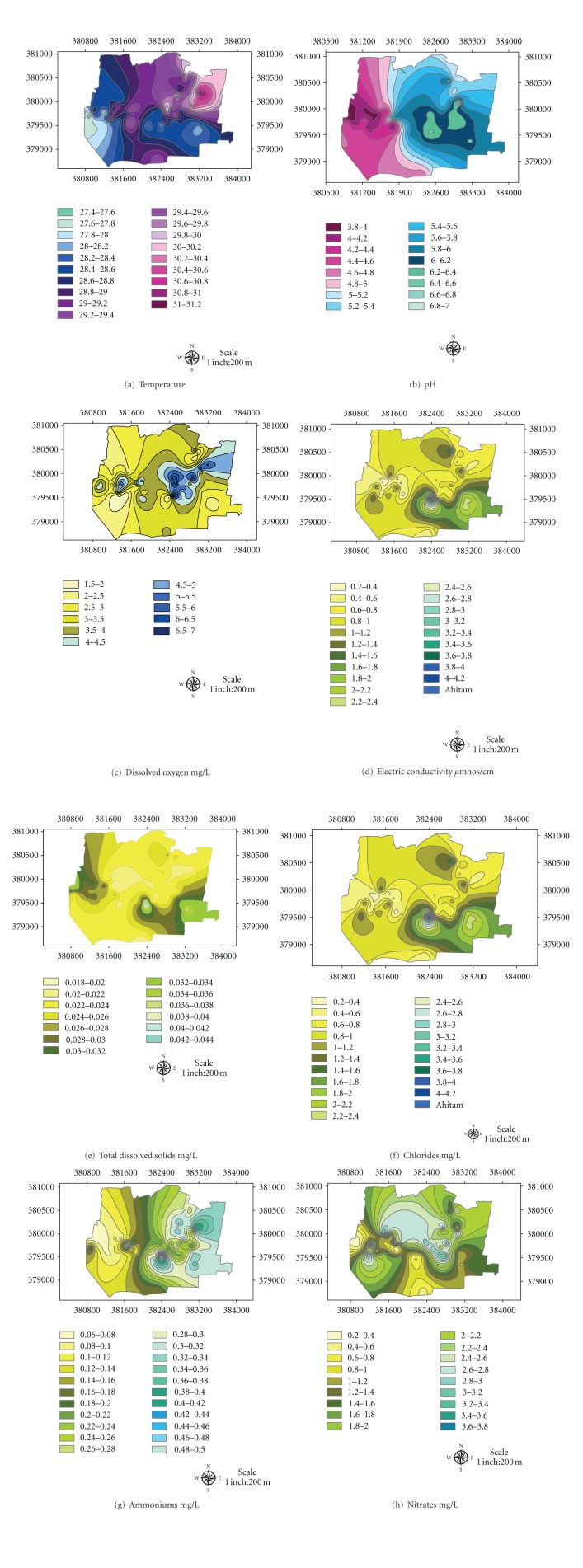
Water quality characterization of the catchment.

The water quality of the study area which includes their physical and chemical characteristics is shown in (Figure 14). The possibility of using these ponds water for livestock, irrigation, and as raw (for washing, bathing, etc.) or portable water was studied, based on standards set by United States Environmental Agency (2008), World Health Organization (2009) (Table 3), and Malaysian Interim Water Quality Standards (2009) (Table 4). Below is the brief description of the water quality parameters observed during the field study.

#### 3.2.1. Colour (TCU) and Turbidity (NTU)

Water colour of the study area has been analysed by TCU (True Colour Units) that shows the colour unit of 9 TCU which is acceptable for raw water. Levels of colour below 15 TCU are usually recommended for raw water, but acceptability may vary. Analyses of Lake A, Lake B, and Lake C of dredged mining operations have turbidity less than 1 NTU, while Lake D created from gravel pit mining operations had higher turbidity values between 2.3 to 11.0 NTU. This pattern is reflective of the grain size of the tailing derived from two different mining methods.

#### 3.2.2. Temperature

The temperature values of the catchment water are shown in (Figure 14(a)). The temperature values ranged 27–30°C. Maxiumum temperature observed in north eastern part of the study area was 31°C. These variations in water temperature can be attributed to the water flow from different parts of the upstream and mixed up together at the downstream part of the catchment. These temperature values are normal according to the climatic conditions of the area.

#### 3.2.3. pH

The pH of water from study area ranged 3–7.0 (Figure 14(b)). The pH of the catchment is highly acidic representing excessive mining operations done in the area. The pH of the eastern part of the study area approaches nearly 7.0. The possible causes of this neutral pH are the formation of wetlands, palm oil plantation, and the dilution factor as water flows downstream. The pH of the study area, however, is quite below the minimum allowable limit for use as raw water (Table 3). The pH of the area tends towards the acidic side. This condition is also related to higher concentration of cations in the water.

#### 3.2.4. Dissolved Oxygen (DO)

The study area has very low dissolved oxygen 1.5–6.5 mg/L (Figure 14(c)). The central part of the study area has dissolved oxygen ranging 3.0–3.5 mg/L, while eastern part of the area has dissolved oxygen ranging 5.0–5.5. These DO values are way below the maximum allowable limit for use as raw water (Table 3).

#### 3.2.5. Electric Conductivity (EC)

Study area of the catchment has a high electric conductivity ranging 3.0–6.5 *μ*s/m only (Figure 14(d)). The central part of the study area has an electric conductivity ranging 3.6–3.8 *μ*s/cm, while eastern part has an electric conductivity 4.6–4.8 *μ*s/m indicating highly dissolved salts and ions in the water.

#### 3.2.6. Total Dissolved Solids (TDSs)

The study area has total dissolved solids ranging from 1800 to 4200 mg/L (Figure 14(e)). The central part of the study area has dissolved oxygen ranging from 2400 to 2600 mg/L, while eastern part of the area has dissolved oxygen ranging from 3000 to 3200 mg/L. These TDS values are way below the maximum allowable limit for use as raw water (Table 3).

#### 3.2.7. Chloride (Cl^−^)

The study area has chlorides ranging from 0.2–4.20 mg/L (Figure 14(f)). The central part of the study area has chlorides ranging from 0.8–1.0 mg/L while eastern part of the area has chlorides concentration ranging from 1.4–1.6 mg/L. The concentration of chloride anions is a way below the maximum allowable limit of 250 mg/L for use as raw water and portable water (Table 3).

#### 3.2.8. Ammonium (NH_4_^+1^)

The study area has ammonium cations concentration ranging from 0.06 to 0.46 mg/L (Figure 14(g)). The central part of the study area has dissolved oxygen ranging from 0.16 to 0.20 mg/L, while eastern part of the area has dissolved oxygen ranging from 0.32 to 0.36 mg/L.

#### 3.2.9. Nitrate (NO_3_^2−^)

The study area has nitrate anions concentration ranging from 0.20 to 3.8 mg/L (Figure 14(h)). The central part of the study area has dissolved oxygen ranging from 2.60 to 2.8 mg/L, while eastern part of the area has dissolved oxygen ranging from 1.4 to 1.6 mg/L. Most of the study area had less than 3.0 mg/L of nitrate anions NO_3_^2−^ recommended allowable limit for raw water.

Comparison with Malaysian Interim Water Quality Standards (INWQS) (Table 4) showed that at all sampling stations colour lies between 5 and 9 TCU so it falls in class I, temperature in normal range, pH class III, electric conductivity falls class III, dissolved oxygen in class III, and total dissolved solids in class III. Acidic pH and low DO are the characteristics of peat swamp water (flowing into the catchment) and also by metal and sand mining activity. The high conductivity values represent high concentration of total dissolved solids. The main source of high TDS value is the recent sand mining activity going on in the study area. This study shows that the water quality is degraded in the area.

### 3.3. Acid Mine Drainage

In the Bestari Jaya catchment, the release of acid mine drainage is still an ongoing environmental issue but by far the largest impact was through the release of particulate mine waste (tailings) which were deposited throughout the catchment.

The main mined ore mineral for tin is the oxide cassiterite (SnO_2_). This mineral is chemically stable; the tin is effectively locked within the mineral and is unlikely to change with time; importantly the tin is not therefore available for uptake by plants or animals. In contrast, other minerals such as pyrite (FeS_2_) and chalcopyrite (CuFeS_2_—the main ore mineral for copper) are sulphides, which can oxidise. In a wet oxygenated environment, pyrite will oxidise to produce sulphuric acid and iron hydroxide as follows [35]:

(2) $$2{\text{FeS}}_{2}+7{\text{O}}_{2}+2{\text{H}}_{2}\text{O}\to 2{\text{FeSO}}_{4}+2{\text{H}}_{2}{\text{SO}}_{4}$$

Bacteria (*Thiobacillus ferrooxidans*) can speed up this chemical process by as much as 100 times:

(3) $$4{\text{FeSO}}_{4}+{\text{O}}_{2}+2{\text{H}}_{2}{\text{SO}}_{4}\to 2\text{Fe}{\left({\text{SO}}_{4}\right)}_{3}+2{\text{H}}_{2}\text{O}$$

The relevance of bacterial catalysis from a treatment perspective is that control of these microbial populations within a mine or spoil heap may significantly limit the potential for pyrite oxidation, and hence the severity of mine water discharges at the surface. Thus, the use of biocides might be a very effective “at source” remediation measure provided they could be successfully and sustainably applied [36].

At pH values of between 4 and 7 the ferric ions produced in this reaction will tend to precipitate out as ochres. Additionally, the sulphuric acid and ferric sulphate produced from pyrite can then oxidise other metal sulphides such as chalcopyrite:

(4) $${\text{CuFeS}}_{2}+\text{Fe}{\left({\text{SO}}_{4}\right)}_{3}\to {\text{CuSO}}_{4}+2{\text{FeSO}}_{4}+2\text{S}$$

If the sulphide minerals are rapidly deposited within the estuarine sediments, which at depth lack free oxygen, then the minerals are chemically stable. If these grains are reworked and exposed to oxygen, then they will start to chemically alter.

In summary, rock mining can result in the release of the following.

1. Metals in solution—the so called acid mine drainage. Some of this metal initially released in solution can be attached through adsorption or absorption onto other mineral surfaces (e.g., on clay minerals).
2. Acid mine drainage can result locally in the precipitation of Fe or Mn ochres which may also contain significant concentrations of other metals.
3. The release of fine grained particulate mine waste including grains of ore minerals which are either locked within larger fragments due to inefficient grinding of the sample during processing or alternatively very fine grains of the ore minerals which are too small to be recovered during mineral processing.
4. The local dumping of coarse mine waste rock piles.
5. Man-made smelt and slag products formed during the metallurgical processing of the ore.

## 4. Conclusion

1. There are total 85 lakes and ponds (14.8%) derived from dredging operations, and the remaining 357 (85.2%) lakes and ponds are the relicts of the gravel pit mining method. About 91.9% (1097) of the ponds derived from the gravel pit method of mining are small, measuring less than 500 m long, but a few (3.3%) are large, measuring between 500 m to 1500 m long. Ponds and lakes derived from the dredging method are generally larger, usually measuring between 300 to 700 m long. Lakes and ponds which are left over from dredging operations generally have a thin layer of sedimentation (varying between 0.1 and 2.6 m) overlaying fine sand, and the depths are not deep (varying between 2 and 3 m). An exception is pond where depth is up to 27 m.
2. Lakes and ponds with limestone bedrock, especially those with exposed outcrops or shallow bed rock will have slightly alkaline pond water with high Ca^2+^ and Mg^2+^. These cations will help to neutralize the surface charges of the suspended clay minerals, hence enabling the clay minerals to settle faster. This explains the observation that almost all the ponds at Bestari Jaya catchment have a clear uppermost layer of supernatant up to 2-3 m.
3. Maximum depth of lakes in the study area is up to 11 m. Most of the lakes that develop from dredging mining operations have average depth lies between 6 and 7 m. Average water discharge from eastern part of the catchment to study area is 0.0418 m^3^/s while from western part of the catchment into the study area is 0.0224 m^3^/s. Average water discharge from the study area of the catchment into Ayer Hitam river is 1.186 m^3^/s.
4. Results show that there is a variation trend of water quality at the catchment. Average values for studied water quality parameters are colour 9–12 TCU, temperature 27–29°C, pH 5–7, conductivity 4-5 S/m, dissolved oxygen 4 mg/L, total dissolved solids 2998 mg/L, while water quality parameters at the inlet of catchment to River Ayer Hitam are colour 5 TCU, temperature 32.19°C, pH 6.47, conductivity 1 S/m, dissolved oxygen 6.59 mg/L, total dissolved solids 2654 mg/L. This shows variation trends at all sampling stations are from upstream to downstream. Possible factors involved in this variation may include formation of wetlands, palm oil plantation, and the dilution factor of water.
5. Based on the results of water quality analysis, catchment water did not meet the standards for use as raw water and portable water, set by US Environmental Protection Agency (2008), World Health organization (2009), and Ministry of Health, Malaysia (2009). Similarly comparison with Malaysian Interim Water Quality Standards (INWQS). At all sampling stations, colour lies between 5 and 9 TCU so it falls in class I, temperature in normal range, pH class III, electric conductivity falls class III, dissolved oxygen in class III, and total dissolved solids in class III. Acidic pH and low DO are the characteristic of peat swamp water (flowing into the catchment) and also by metal and sand mining activity. The high conductivity values represent high concentration of total dissolved solids. The main source of high TDS value is the recent sand mining activity going on in the study area. This study shows that the water quality is degraded in the area.
6. In the Bestari Jaya catchment, the release of acid mine drainage is still an ongoing environmental issue but by far the largest impact was through the release of particulate mine waste (tailings) which were deposited throughout the catchment.

## Figures and Tables

**Table 1:** Lakes develop after different mining operations [33].

Mukim	Dredged lakes	Dredged sedimentation lakes	Gravel pump mine pits	Open cast tailings	Total
Berjuntai	273	27	167	108	442

**Table 2:** Characteristics of the studied lakes at Bestari Jaya catchment.

Lake	Survey date	Type of lake	Maximum length (m)	Maximum width (m)	Average thickness of sediments (m)	Side slopes	Estimated volume of sediments (m^3^)	Surroundings nature	Present use	Water depth sides (m)	Water depth centre (m)
Lake A	20/06/2009	Dredged	1625	875	1.0	Steep	14500	Undulating with thin grass cover	Waste land	4.0-5.0	10-11
Lake B	07/09/2009	Dredged	1354	689	0.82	Moderate	12355	Undulating with thin grass cover	Waste land	2.0-3.0	9.0-10.0
Lake C	02/11/2009	Dredged	1273	791	0.97	Gentle	13764	Undulating with thin grass cover	Waste land	1.0-2.0	6.0-7.0
Lake D	04/01/2009	Palong mine pit	1485	804	0.74	Steep	12355	Undulating with thin grass cover	Waste land	0.5–1.0	4.0-5.0

**Table 3:** Maximum allowable limit for raw water and portable water.

Serial number	Parameter	Raw untreated water (after Ministry of Health, Malaysia)	Portable water	
			Ministry of Health, Malaysia (2009)	US Environmental Protection Agency (2008) and World Health organization (2009)
1	Physical characteristics			
	Turbidity	1000 NTU	5 NTU	5 NTU
	Colour	300 Hazen	15 Hazen	5 Hazen
	pH	5.5–9.0	6.5–9.0	6.8–9.2

2	Inorganic			
	Dissolved solids	1500 mg/L	100 mg/L	500 mg/L
	Total solids	—	—	1500 mg/L
	Chlorides	250 ppm	250 ppm	250 ppm
	Nitrate	10 ppm	10 ppm	45 ppm
	Iron Fe	1.0 ppm	0.3 ppm	0.3 ppm
	Fluoride	1.5 ppm	0.9 ppm	1.5 ppm
	Ammonia N	0.5 ppm	0.5 ppm	—
	Total nitrogen NO_3_	1.0 ppm	—	—
	Hardness	500	500	—
	Sulphate	400 ppm	400 ppm	400 ppm

3	Elements/compounds			
	Mercury	0.001 ppm	0.001 ppm	0.002 ppm
	Cadmium	0.005 ppm	0.005 ppm	0.01 ppm
	Selenium	0.01 ppm	0.01 ppm	0.01 ppm
	Arsenic	0.05 ppm	0.05 ppm	0.05 ppm
	Cyanide	0.1 ppm	0.1 ppm	0.05 ppm
	Lead	0.1 ppm	0.05 ppm	0.05 ppm
	Chromium	0.05 ppm	0.05 ppm	0.05 ppm
	Silver	0.05 ppm	0.05 ppm	0.05 ppm
	Copper	1.0 ppm	1.0 ppm	1.0 ppm
	Magnesium	150 ppm	150 ppm	—
	Manganese	0.2 ppm	0.1 ppm	0.05 ppm
	Zinc	5.0 ppm	5.0 ppm	5.0 ppm
	Sodium	200 ppm	200 ppm	200 ppm
	Aluminium	—	0.2 ppm	0.2 ppm
	Oil and grease	0.3 ppm	0.3 ppm	0.3 ppm
	Phenol	0.002 ppm	0.002 ppm	—

Recommended concentration limits for these constituents are mainly to provide acceptable aesthetic and taste characteristic.

**Table 4:** Interim National Water Quality Standards for Malaysia (INWQS) (2008).

Parameters		Classes					
	Unit	I	IIA	IIB	III	IV	V
Ammonical nitrogen	mg/L	0.1	0.3	0.3	0.9	2.7	>2.7
BOD	mg/L	1	3	3	6	12	>12
COD	mg/L	10	25	25	50	100	>100
DO	mg/L	7	5–7	5–7	3–5	<3	<1
pH		6.5–6.8	6–9	6–9	5–9	5–9	—
Colour	TCU	15	150	150	—	—	—
Electric conductivity*	*μ*mhos/cm	1000	1000	—	—	6000	—
Floatables		N	N	N	—	—	—
Odour		N	N	N	—	—	—
Salinity	%	0.5	1	—	—	2	—
Taste		N	N	N	—	—	—
Total dissolved solids	mg/L	500	1000	—	—	4000	—
Total suspended solids	mg/L	25	50	50	150	300	300
Temperature C	°C	—	Normal + 2°C	—	Normal + 2°C	—	—
Turbidity NTU	NTU	5	50	50	—	—	—
Faecal coliform**	Counts/100 mL	10	100	400	5000 (20000)a	5000 (20000)a	—
Total coliform	Counts/100 mL	100	5000	5000	50000	50000	>50000

Class I: Conservation of natural environment water supply—practically no treatment necessary. Fishery 1: very sensitive aquatic species.

Class IIA: Water Supply II—conventional treatment required. Fishery ll: sensitive aquatic species. CLASS IIB: recreational use with body contact.

Class III: Water Supply III—extensive treatment required. Fishery lll: common, of economic value, and tolerant species livestock drinking.

Class IV: Irrigation.

*: Related parameters, only one recommended for use.

**: Geometric mean.
